# Dermatomyositis, Rhabdomyolysis, and Statin-Induced Myopathy: A Case Report Illustrating the Spectrum of Myopathy

**DOI:** 10.7759/cureus.84854

**Published:** 2025-05-26

**Authors:** Htet Zaw Lin, Yair Oo, Sai Laung Khay, Ahmar Ijaz, Vui H Chong, Jackson Tan

**Affiliations:** 1 Department of Nephrology, Raja Isteri Pengiran Anak Saleha Hospital, Bandar Seri Begawan, BRN; 2 Department of Medicine, Raja Isteri Pengiran Anak Saleha Hospital, Bandar Seri Begawan, BRN

**Keywords:** dermatomyositis, furosemide-induced rhabdomyolysis, idiopathic inflammatory myopathies, intravenous immunoglobulin therapy, proton pump inhibitor induced, rhabdomyolysis causing acute kidney injury, statin-induced rhabdomyolysis

## Abstract

This report describes a rare presentation of rhabdomyolysis and dermatomyositis in a patient with existing statin-induced myopathy, presenting with acute-on-chronic kidney disease. The patient had an initial diagnosis of statin-induced myopathy through mild creatinine kinase elevation following the prescription of atorvastatin. However, eight months after this, she presented with fulminant rhabdomyolysis following the introduction of furosemide and omeprazole. Dermatomyositis was diagnosed concurrently through a clinical presentation of heliotrope rash and proximal muscle weakness, which was confirmed by muscle biopsy. As the patient was already in advanced kidney failure with features of fluid overload, aggressive treatment with intravenous fluids was withheld. After failing to respond to high-dose steroids, the patient was treated early with intravenous immunoglobulins, which resulted in a rapid drop in creatine kinase level, improvement of muscle weakness, and renal function.

This study depicts the nuanced and idiosyncratic spectrum of myopathy through a progressive transition of symptoms over a defined timeframe. It also highlights the adverse interactions between proton pump inhibitors, diuretics, and statins in triggering rhabdomyolysis and unmasking the presentation of dermatomyositis. Furthermore, it demonstrates the therapeutic effects of early intervention with intravenous immunoglobulin as rescue therapy for rhabdomyolysis in a situation where conventional fluid resuscitation was limited by advanced kidney failure.

## Introduction

Idiopathic inflammatory myopathies (IIM), collectively known as myositis, are heterogeneous disorders characterized by muscle weakness and muscle inflammation. The most common subgroups in adults are dermatomyositis, polymyositis, inclusion body myositis, and immune-mediated necrotizing myositis [1]. In addition, idiopathic inflammatory myopathies can also overlap with renal autoimmune conditions like systemic lupus erythematosus, systemic sclerosis, and glomerulonephritis, presenting with features of kidney injury [2]. Rhabdomyolysis, a medical condition involving the breakdown of muscle tissue, is known to be associated with idiopathic inflammatory myopathies with an estimated incidence of 20% [3,4]. However, most reported cases of rhabdomyolysis are seen in polymyositis, with very few published case reports associated with dermatomyositis [5].

Rhabdomyolysis can often be triggered by certain medications and medical conditions [6]. This can happen when a patient is already on statin therapy; however, occasionally, interactions with other medications can intensify the statin-induced muscle breakdown [7]. Rarely, the combinations of different drugs can lead to the new development of idiopathic inflammatory myopathies, independent of the effects of rhabdomyolysis [8,9].

This case report describes a unique presentation of acute rhabdomyolysis and new-onset dermatomyositis in a patient with advanced kidney disease from a potential trigger of concomitant usage of statin, furosemide, and proton pump inhibitor. The report also highlights the beneficial effects of intravenous immunoglobulin in anuric patients, which will usually preclude aggressive fluid interventions in rhabdomyolysis.

## Case presentation

A 56-year-old lady presented to our tertiary care hospital with a short history of generalized body weakness, fluid retention, and shortness of breath in early February 2025. She had a history of long-standing type 2 diabetes mellitus (T2DM) complicated by retinopathy and nephropathy. Her baseline serum creatinine function was around 200-300 mmol/L at the beginning of 2024.

Prior to this admission, several key events occurred in the preceding months. She was started on atorvastatin 20 mg in an outpatient clinic for hypercholesterolemia in April 2024. A creatine kinase level (CK) after commencement of statin was slightly elevated at 525 U/L, indicative of asymptomatic statin-induced myopathy, but there was no evidence of cardiac ischemia on routine investigations. Following that, she had an acute coronary syndrome event in early December 2024, which necessitated intervention with percutaneous coronary angioplasty to her right coronary artery. She had intravenous contrast exposure, which resulted in her serum creatinine deteriorating to around the 600-700 mmol/L range post-angiogram. She was re-admitted to the hospital in late December 2024 with increasing fluid retention and elevated creatine kinase of 1246 U/L. However, a new coronary event was excluded, and she was discharged with an increased dose of statin (rosuvastatin 40 mg), omeprazole, and furosemide. Atorvastatin was swapped with rosuvastatin as the latter was thought to be less myopathic.

**Figure 1:**
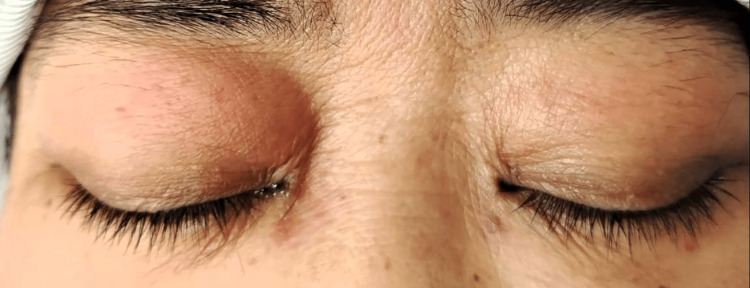
Heliotrope rash around eyes.

At the time of her admission in February 2025, her serum creatinine and creatine kinase were noted to be 1239 mmol/L and 18654 U/L, respectively. She had oligo-anuria with dark-red urine in the collection samples. Urine myoglobin was detected, and she was subsequently diagnosed with rhabdomyolysis, with cessation of statin therapy. Potassium and calcium remained in the normal range, although phosphate was moderately high, in keeping with chronic kidney disease. However, the patient was initiated on dialysis due to worsening urine output within a week of admission. There was liver enzyme derangement (ALT - seven times the normal range), likely in keeping with rhabdomyolysis. She was evaluated by neurologists for quadriparesis, which revealed weakness in all four limbs, more evident in the proximal than distal limbs, with signs consistent with myopathy. The upper limb proximal and distal muscle power were 1/5 and 4/5, respectively, while the lower limb proximal and distal muscle weakness were 2/5 and 4/5, respectively. There was also weaker neck flexion compared to extension, which resulted in her having difficulty holding her head in an upright position. All the reflexes were normal. There was a heliotrope rash around her eyes (Figure 1). However, Gottron’s papules were not seen. Apart from shortness of breath from fluid overload, there was no other symptom suggestive of other systemic organ involvement.

Routine autoimmune investigations revealed no autoantibodies including antinuclear antibody (ANA), extractable nuclear antigen (ENA), antineutrophil cytoplasmic antibody (ANCA), anti-Sjögren's syndrome-related antigen A (anti-Ro), anti-Sjögren's syndrome-related antigen A (anti-SSA), anti-nucleosome remodeling deacetylase complex antibody (anti-Mi-2), anti-melanoma differentiation-associated gene 5 antibody (anti-MDA5), anti-nuclear matrix protein 2 antibody (anti-NXP2), anti-small ubiquitin-like modifier activating enzyme antibody (anti-SAE), and anti-3-hydroxy-3-methylglutaryl-coenzyme A reductase antibody (anti-HMGCR). Myositis-specific and myositis-associated antibodies, including a subtype of SSA antibodies (anti-Ro52), anti-isoleucyl-tRNA synthetase (OJ), anti-glycyl-tRNA synthetase (EJ), anti-alanyl-tRNA synthetase (PL-12), anti-signal recognition particle (SRP), anti-histidyl-tRNA synthetase (Jo-1), anti-PM/Scl complex, 75 kDa subunit (PM/Scl-75), anti-PM/Scl complex, 100 kDa subunit (PM/Scl-100), Ku (blot) (anti-Ku antigen {DNA repair protein}), anti-small ubiquitin-like modifier activating enzyme 1 (SAE1), anti-nuclear matrix protein 2 (also known as MORC3) (NXP2), anti-melanoma differentiation-associated gene 5 (also known as CADM-140) (MDA5), anti-transcription intermediary factor 1-gamma (also called p155/140) (TIF1-γ), anti-Mi-2 beta (part of nucleosome remodeling complex) (Mi-2β), and anti-Mi-2 alpha (same complex as Mi-2β) (Mi-2α) were all negative. Other routine blood investigations included tumor marker screening, and hematological and liver profiles were normal. Additional investigations included chest x-ray showing left-sided pleural effusion and interstitial edema. Computed tomography scan of thorax, abdomen, and pelvis did not show any significant abnormalities apart from pleural effusion. Nerve conduction study showed findings consistent with mononeuritis multiplex, with moderate prolongation of median latency and moderately reduced compound muscle action potentials (CMAPs). Ulnar and fibular nerve conduction studies showed similar reductions in CMAPs. Fibular conduction showed marked reduction of the compound muscle action potential (CMAP). Electromyography study revealed runs of fibrillation potentials and positive sharp waves with occasional runs of pseudo-myotonic discharges in keeping with a necrotic myopathic process. Muscle biopsy from vastus medialis muscle showed evidence of necrosis, patchy atrophy, and degenerated fibers. Immunohistochemical stain highlighted patchy, scattered T-lymphocytes and histiocytes but there was no significant endomysial or perimysial fibrosis in trichrome staining. The final diagnosis was in keeping with a diagnosis of acute inflammatory myopathy, consistent with dermatomyositis or polymyositis. Further analysis through the online European League Against Rheumatism/American College of Rheumatology (EULAR/ACR) calculator revealed a definite idiopathic inflammatory myopathy (IIM) diagnosis, with a subgroup classification of dermatomyositis [1].

**Figure 2:**
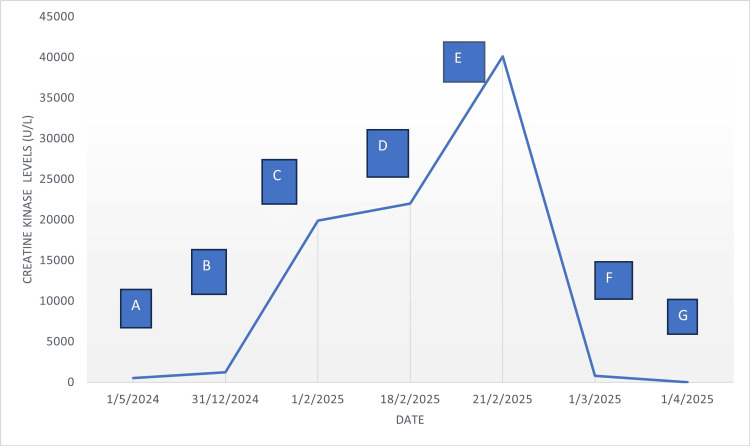
Trend of CK level against time with specific key events. CK: creatine kinase

During the ensuing days of hospitalization, the patient developed progressive reduction of her urine output and required hemodialysis treatment within four days of admission. However, treatment for rhabdomyolysis was limited by hypervolemia and intravenous fluid was restricted. A kidney biopsy was not performed as it was felt that she already had advanced chronic kidney disease and reversibility of kidney disease was unlikely. Over the course of the next week, there was no improvement in her muscle weakness and blood creatine kinase levels, despite maximal therapy with methylprednisolone. She also had a lower gastrointestinal bleed from rectal ulcerations, which required colonoscopic intervention to stop the bleeding. A decision was made to initiate intravenous immunoglobulin therapy early due to a lack of physical improvement and ongoing muscle injury. She made a quick recovery following intravenous immunoglobulin treatment with improvement of creatine kinase level, urine output, and renal solute clearance. Figure 2 and Table 1 illustrate the creatine kinase trends over time and the key events that have influenced the trends.

**Table 1:** Codes for Figure 2 and summary of key events. CK: creatine kinase; IV: intravenous; IVIG: intravenous immunoglobulin

Code	Key events description	Time frame	CK levels	Diagnosis
A	After commencement of atorvastatin 20 mg	May 2024	525	Possible statin-induced myopathy
B	Atorvastatin increased to 40 mg	Dec 2024	1,246	Statin-induced myopathy
C	30 days after rosuvastatin 40 mg, omeprazole 20 mg, and furosemide 160 mg	Jan 2025	19,901	Rhabdomyolysis from drug interactions with statins
D	IV methylprednisolone 500 mg daily for 6 days was started	Feb 2025	22,000	Dermatomyositis
E	IV immunoglobulins were initiated due to increment in CK levels and non-resolution of symptoms	Feb 2025	40,127	Refractory dermatomyositis refractory to steroid treatment
F	Drastic reduction of CK levels 10 days after completion of IVIG	Feb 2025	786	Dermatomyositis and rhabdomyolysis responding to IVIG
G	Normalization of CK levels	April 2025	17	Recovery of renal function

## Discussion

Clinical spectrum of myopathy

This unique case report presents overlapping features of several clinical phenotypes of myopathy - statin-induced myopathy, dermatomyositis, and rhabdomyolysis. Another similar condition, statin-induced immune-mediated necrotizing myopathy (IMNM), was excluded by the absence of anti-3-hydroxy-3-methylglutaryl-coenzyme A reductase (anti-HMGCR) antibody. The clinical presentation of the patient seems to have evolved from asymptomatic statin-induced myopathy to rhabdomyolysis and dermatomyositis within a temporal timeframe that was punctuated by potential triggers. Statin-induced myopathy is usually a self-limiting condition that occurs in up to 30% of patients taking statins, and generally with non-specific muscle aches and minimal or no increment in creatine kinase [10]. Dermatomyositis is an immune-mediated disorder characterized by skin rash and muscle weakness; however, hallmark features include heliotrope rash, Gottron’s papules, nailfold capillary dilatation, and photosensitivity [11]. Diagnosis can be confirmed through the European League Against Rheumatism/American College of Rheumatology (EULAR /ACR) classification, which utilizes clinical, laboratory, and muscle biopsy criteria to calculate the probability of idiopathic inflammatory myopathies (IIM) and their subclassifications like dermatomyositis [1]. Rhabdomyolysis is a medical condition that causes necrosis of damaged skeletal muscle tissue, leading to release of intracellular components into the systemic circulation, including myoglobin, creatine kinase, and other muscle enzymes [7]. This is usually caused by trauma or direct muscle injury, but underlying muscle disorders and medications are well-known. Our patient, who had statin-induced myopathy at the outset, also fulfilled the European League Against Rheumatism/American College of Rheumatology (EULAR/ACR) criteria for definite idiopathic inflammatory myopathies (IIM) with a subgroup classification of dermatomyositis. The presence of characteristic proximal muscle weakness, heliotrope rash, and muscle biopsy findings was in keeping with the diagnosis of dermatomyositis [1]. Rhabdomyolysis was confirmed by the characteristic creatine kinase (CK) rise and clinical presentation of dark urine and presence of myoglobinuria. Table 2 shows the different clinical presentations of the three aforementioned conditions.

**Table 2:** Comparison of demographic and clinical factors in statin-induced myopathy, rhabdomyolysis and dermatomyositis. CK: creatine kinase; DM: dermatomyositis; MRI: magnetic resonance imaging; SNP: single nucleotide polymorphism; SLCO1B1: solute carrier organic anion transporter family member 1B1; Jo-1: histidyl-tRNA synthetase antibody; Mi-2: nucleosome remodeling deacetylase complex antibody; NXP-2: nuclear matrix protein 2 antibody; TIF1-γ: transcriptional intermediary factor 1-gamma antibody; MDA5: melanoma differentiation-associated gene 5 antibody; SAE: small ubiquitin-like modifier activating enzyme antibody

Variables	Self-limiting statin myopathy (myalgia/non-immune myositis) [10,12]	Rhabdomyolysis [10,13]	Dermatomyositis [11,14]
Incidence	7-29% of statin users	0.3-13.5/1,000,000 users (0.0003-0.0013%)	3.98 and 4.68 per 1,000,000
Age range	Increased risk with age	Median age: 64 (SD: 14) years. Increased risk with age	Between 40 and 50 years
Gender predisposition	Female	Male (70%)	Female
Myalgia	Common	Common and painful. Associated with myoglobinuria. Acute renal failure	Common and associated with skin rash (typically Gottron papules and heliotrope rash)
Proximal muscle weakness	Uncommon	Common	Common, often associated with esophageal muscle weakness and dysphagia
Genetic risk factor/autoantibodies	SNP in SLC01B1	SNP in SLC01B1	Myositis specific antibodies like anti-Jo-1, anti-Mi-2, anti-NXP-2, anti-TIF1-y, anti-MDA5, and anti-SAE
Diagnosis	Clinical muscle biopsy not required	Marked elevation of CK. Muscle biopsy not required	Characteristic skin findings and muscle biopsy
Muscle biopsy	Variable findings	Muscle necrosis	Muscle necrosis with inflammation and perifascicular atrophy
MRI	Normal may show muscle edema	Diffuse swelling of affected muscle groups. Areas of breakdown with liquefied necrosis	Patterns of muscle edema, especially in proximal muscles, are bilateral and symmetrical. Honeycomb or reticular pattern is a hallmark of DM
Management	Withdrawal of statin	Withdrawal of trigger agents and hydration	Withdrawal of trigger agents and immunosuppressive therapy
Outcome	Self-limiting	Self-limiting	Can be progressive

Pathophysiological mechanisms

We believed that a shared pathophysiological mechanism has shaped the overall case presentation, as demonstrated by the overlapping clinical features from the myopathy spectrum. Statins inhibit 3-hydroxy-3-methylglutaryl-coenzyme A (HMG-CoA) reductase, the rate-limiting enzyme in cholesterol synthesis, which also impacts coenzyme Q10 (CoQ10) production [15]. Coenzyme Q10 (CoQ10) is essential for cell signaling, mitochondrial function, and membrane integrity, with deficiencies increasing susceptibility to muscle injury [15]. In the present case, there could be accelerated depletion of coenzyme Q10 (CoQ10) with increased dosage of statin, which was further exacerbated by co-administration of facilitators like furosemide and omeprazole that increase muscle cells' vulnerability to injury through membrane desensitization and oxidative stress. The combined effects of these would have accelerated the development of rhabdomyolysis with muscle cell necrosis and release of muscle contents into the circulation. Dermatomyositis, on the other hand, is characterized by an immune-mediated mechanism that primarily affects muscle capillaries, causing small vessel ischemia, rather than direct muscle injury. There is a known relationship between dermatomyositis and type 1 diabetes mellitus, which was thought to be related to shared genetic autoimmunity risks [16]. In the present case, the patient also had long-standing type 2 diabetes mellitus, which may contribute to chronic inflammation and perpetuate immunological and inflammatory pathways, aggravating underlying muscle injuries.

Association of dermatomyositis and rhabdomyolysis

This case has identified a few unique educational elements as follows: concomitant occurrence of dermatomyositis and rhabdomyolysis, drug interactions-induced rhabdomyolysis, and benefits of intravenous immunoglobulin in anuric rhabdomyolysis. Firstly, it adds to the limited literature on concurrent rhabdomyolysis and dermatomyositis, which has been recognized as a pathological association since the 1970s, with sporadic reports in the literature in the ensuing decades [17]. The underlying pathophysiological mechanism explaining the association could be the innate inflammation and muscle necrosis present through T-cell autoimmune-mediated injury to the muscle fibers in patients with dermatomyositis. Speculatively, the persistent inflammatory condition can compound or exacerbate any additional injury or insult (through medications, infections, or trauma) to the muscle and predispose to rhabdomyolysis. Dermatomyositis differs from polymyositis because the autoimmune pathology in the former affects the small blood vessels in the muscles, leading to ischemic damage rather than directly causing muscle necrosis.

Our literature review identified only 10 cases with adequate descriptive information from a diverse number of patients from different ethnic backgrounds and continents [18-27]. Most of the patients were young (under 60 years of age) and had no specific trigger for dermatomyositis and rhabdomyolysis. Four out of the 10 cases were treated with intravenous immunoglobulin with successful outcomes [19-21,25], and some cases have additional extra-systemic neurological and gastrointestinal involvement [20,24,25]. Fukunaga et al. described a case similar to ours, in which the patient also had gastrointestinal involvement, and the symptoms of dermatomyositis resolved after treatment with intravenous immunoglobulin [25]. Indeed, two other case reports of dermatomyositis (without rhabdomyolysis) showed coincidental gastrointestinal complications like gastrointestinal ulcers and duodenal perforation that resolved after treatment with intravenous immunoglobulin [28,29]. Interestingly, despite the presentation of rhabdomyolysis with myoglobinuria, many cases did not report the presence of acute kidney injury [19,22,23,26]. Additionally, there are also reports of statin-induced dermatomyositis, without rhabdomyolysis, indicating that statins can present as an independent immunological trigger without manifesting with gross muscle disorders [30-32]. Table 3 summarizes the studies reporting concurrent presentations of these two disease entities.

**Table 3:** Summary of case reports with dermatomyositis and rhabdomyolysis. CK: creatine kinase; IV MP: intravenous methylprednisolone; IVIG: intravenous immunoglobulin; anti-Mi-2: anti-nucleosome remodeling deacetylase complex antibody; anti-TIF1-γ: anti-transcriptional intermediary factor 1-gamma antibody; anti-NXP-2: anti-nuclear matrix protein 2 antibody; anti-SAE: anti-small ubiquitin-like modifier activating enzyme antibody; anti-HMGCR: anti-3-hydroxy-3-methylglutaryl-coenzyme A reductase antibody; EMG: electromyography; IV: intravenous

Case	One [18]	Two [19]	Three [20]	Four [21]	Five [22]	Six [23]	Seven [24]	Eight [25]	Nine [26]	Ten [27]
Origin	India	Japan	USA	Bangladesh	USA	USA	USA	Japan	Greece	Belgium
Age/sex	20/F	24/M	30/M	49/F	41/F	64/F	55/M	31/M	25/F	70/F
Muscle pain/weakness	Right calf pain and proximal muscle weakness	Thigh pain and proximal muscle weakness	Generalized weakness and proximal quadriparesis	Proximal muscle weakness with quadriparesis	Generalized pain and proximal pattern of weakness	Mainly lower limb pain with bilateral lower limb weakness	Left leg pain and weakness of dorsiflexion of left leg	Weakness of lower limbs	Upper and lower limb pain with proximal muscle weakness	Back pain and bilateral upper and lower limbs
Heliotropic rash	Not mentioned	Present	Not mentioned	Not mentioned	No	Not mentioned	Present	Not mentioned	Not mentioned	Not mentioned
Peak CK levels U/L	28,950	40,700	49,548	19,000	129,830	4,100	11,900	16,950	3,974	7,780
Myositis antibodies	Not mentioned	Negative	Anti-Mi-2 positive	Anti-Mi-2 positive	Anti-TIF-1y positive	Anti-NXP-2 positive	Not mentioned	Anti-nuclear antibody positive	Negative	Anti-NCP (-2)
Muscle biopsy	Extensive myonecrosis and active rhabdomyolysis	Cross striations were present in most fibers, and fragmentation of the sarcoplasm was observed in some fibers. Inflammatory infiltrates, myofiber necrosis, or regeneration were not observed	Spindle cells and round cells infiltrating skeletal muscles with perifascicular atrophy were suggestive of proliferative inflammatory myositis with muscle necrosis	Large necrosis and phagocytosis. Localized necrotic fiber in other area. Electromyogram showed spontaneous fibrillations, decreased amplitude in the Deltoid and Iliopsoas muscles	Normal biopsy	Myofiber atrophy with endomysial macrophage infiltration	Not done. Diagnosis based on presence of skin changes by dermatologists	Prominent necrotic and regenerating changes in muscle fibers and clustered inflammatory cells in endomysial and perivascular sites	Focal necrosis and acute inflammation of muscle fibers. Mild perivascular mononuclear infiltration	Normal biopsy
Steroid treatment (methylprednisolone {MP} or oral steroids)	IV MP followed by oral	IV MP	IV MP followed by oral	Oral	IV MP	Oral	Oral	IV MP followed by oral	IV MP followed by oral	Oral
Additional immunosuppression	No	IVIG	IVIG and mycophenolate mofetil	Azathioprine IVIG	-	-	-	Plasmapheresis and IVIG	No	No
Renal outcome	Acute kidney injury without needing dialysis	Normal renal function but significant proteinuria was seen at presentation	Not discussed	No acute kidney injury	No acute kidney injury	Acute kidney injury without needing dialysis	Not discussed	Not discussed	No acute kidney injury	Acute kidney injury needing dialysis, with recovery of renal function after one week
Other systemic associations	-	-	Acute inflammatory demyelinating polyneuropathy in an Epstein-Barr virus-infected patient	-	-	-	Unilateral foot drop	Megaduodenum with impaired gastric and duodenal emptying	Pregnancy and fetal loss	-

Rhabdomyolysis from drug interactions

Another interesting feature of this case was the serious iatrogenic complications of statin-induced rhabdomyolysis through interactions with different drugs. Usage of statin is associated with a 60-fold increase in rhabdomyolysis incidence compared to those not on statin [7]. The Food and Drug Administration (FDA) Adverse Event Reporting System reported that furosemide and proton pump inhibitors are the two most common drug-drug interactions (DDI) causing rhabdomyolysis, with simvastatin, rosuvastatin, and atorvastatin as the most common statins involved with these interactions [33]. Furosemide, by causing electrolyte imbalances, can restrict blood flow to muscle groups and synergistically increase the risk of rhabdomyolysis when used with lipophilic statins, compounding muscle injury and exacerbating hypokalemia-induced muscle paralysis [34]. Proton pump inhibitor (PPI), on the other hand, can inhibit cytochrome P450 3A4 (CYP3A4), which mediates the breakdown of statin, leading to accumulation and greater myotoxic effects [35]. Mitsuboshi et al. compared the usage of proton pump inhibitors (PPIs) or histamine 2 receptor antagonists in patients on statins or fibrates and found that patients on proton pump inhibitors (PPIs) were more likely to have rhabdomyolysis, while those on histamine receptor antagonists demonstrated no increased risk [36]. A few possible mechanisms have been postulated. The first one suggested that proton pump inhibitors selectively bind to H+/K+ -ATPase in stomach parietal cells, which causes alterations in intracellular pH that increase susceptibility to cellular degradation and risk of rhabdomyolysis [37]. Another hypothesis speculated that the proton pump inhibitor (PPI) may predispose to autoimmune disease, leading to immunologically mediated injuries to muscles [38]. The latter hypothesis also lends to a case report by Jakubowski et al., which showed that omeprazole can independently cause rhabdomyolysis and polymyositis, without the effects of statin [8]. In the present case, the coincidental timings of the initiation of furosemide, omeprazole, and high-dose statin fit the temporal pattern of creatine kinase increase and clinical presentation of rhabdomyolysis and dermatomyositis (Figure 2).

Beneficial effects of intravenous immunoglobulin (IVIG)

Lastly, we want clinicians to be cognizant of the effects of intravenous immunoglobulin (IVIG) in similar scenarios. The patient had acute anuric kidney injury, likely related to direct myoglobin damage to the kidney through renal medullar vasoconstriction and direct tubular cytotoxic effect from intratubular myoglobin cast formation. Conventional treatment of rhabdomyolysis with aggressive fluid resuscitation was not possible due to poor urine output and fluid overload status. The disease was also recalcitrant to conventional treatment with high-dose steroids, and the patient suffered from gastrointestinal complications of steroids, which necessitated the early, fortuitous usage of intravenous immunoglobulin (IVIG). Intravenous immunoglobulin (IVIG), through its immunomodulatory effects, reduces inflammation, autoantibody activity, and decreases tissue damage. Dalakas et al. performed a randomized controlled study that showed that intravenous immunoglobulin (IVIG) is a safe and effective treatment, with the vast majority of patients with dermatomyositis showing clinical benefits, while those who continued on standard of care steroid doses had inferior outcomes [39]. Mizoguchi et al. reported a case of dermatomyositis with rhabdomyolysis and acute kidney injury (AKI) treated successfully with intravenous immunoglobulin (IVIG), having failed treatment with conventional steroids [19]. In the present case, early usage of intravenous immunoglobulin (IVIG) has reduced the damage caused by rhabdomyolysis and improved the kidney outcome of the patient.

## Conclusions

This case illustrates how the clinical presentation traverses the clinical phenotype spectrum of myopathy. It also adds to the limited body of evidence on concurrent rhabdomyolysis and new-onset dermatomyositis. We want to highlight the importance of pharmacovigilance, especially when evaluating the iatrogenic causes of rhabdomyolysis due to interactions of statins with many potential drugs. Early intervention with intravenous immunoglobulin (IVIG) limits the impact of kidney injury from myoglobin through progressive muscle damage and enables salvage of kidney function.
